# Analysis of codon usage bias of envelope glycoprotein genes in nuclear polyhedrosis virus (NPV) and its relation to evolution

**DOI:** 10.1186/s12864-016-3021-7

**Published:** 2016-08-24

**Authors:** Yongchao Zhao, Hao Zheng, Anying Xu, Donghua Yan, Zijian Jiang, Qi Qi, Jingchen Sun

**Affiliations:** 1 Subtropical Sericulture and Mulberry Resources Protection and Safety Engineering Research Center, Guangdong Provincial Key Laboratory of Agro-animal Genomics and Molecular Breeding, College of Animal Science, South China Agricultural University, Guangzhou, 510642 People’s Republic of China; 2 Sericultural Research Institute, Chinese Academy of Agricultural Sciences, Zhenjiang Jiangsu, 212018 People’s Republic of China

**Keywords:** Nuclear polyhedrosis virus, Codon usage bias, Envelope glycoprotein, Evolution

## Abstract

**Background:**

Analysis of codon usage bias is an extremely versatile method using in furthering understanding of the genetic and evolutionary paths of species. Codon usage bias of envelope glycoprotein genes in nuclear polyhedrosis virus (NPV) has remained largely unexplored at present. Hence, the codon usage bias of NPV envelope glycoprotein was analyzed here to reveal the genetic and evolutionary relationships between different viral species in baculovirus genus.

**Results:**

A total of 9236 codons from 18 different species of NPV of the baculovirus genera were used to perform this analysis. Glycoprotein of NPV exhibits weaker codon usage bias. Neutrality plot analysis and correlation analysis of effective number of codons (ENC) values indicate that natural selection is the main factor influencing codon usage bias, and that the impact of mutation pressure is relatively smaller. Another cluster analysis shows that the kinship or evolutionary relationships of these viral species can be divided into two broad categories despite all of these 18 species are from the same baculovirus genus.

**Conclusions:**

There are many elements that can affect codon bias, such as the composition of amino acids, mutation pressure, natural selection, gene expression level, and etc. In the meantime, cluster analysis also illustrates that codon usage bias of virus envelope glycoprotein can serve as an effective means of evolutionary classification in baculovirus genus.

**Electronic supplementary material:**

The online version of this article (doi:10.1186/s12864-016-3021-7) contains supplementary material, which is available to authorized users.

## Background

Codons are not used equally in most organisms. During an organisms evolutionary history, preference for using a particular synonymous codon will be formed within a species or gene in the long-term. Codons which are used in higher frequency within species or genes are referred to as optimal codons. Codon usage bias itself refers to such cases in which codons are utilized in higher frequency than other synonymous codons during the process of translation, often as a result of adaptive evolution [1]. Analysis of codon usage bias is thus of vital significance in the quest to improve exogenous gene expression levels within host cells. Codon bias analysis is a common phenomenon in many species, such as *Escherichia coli* [2], *Arabidopsis thaliana* [3], *Xanthophyllomyces dendrorhous* [4], *Taenia saginata* [5], *Megalobrama amblycephala* [6], metazoans [7], and even human beings [8]. Recent studies have shown that the employment of some special synonymous codons can affect protein folding as well as errors in folding [9, 10]. Furthermore, studies have shown that the inherent links between codon usage and thus amino acids influence the protein components of cells [11]. At the same time, thoroughly understanding codon usage bias plays a central role in making accurate prediction of related gene functions.

Different genes exhibit different codon usage bias in the same genome. Mutation, natural selection, and random drift were the three major factors for species’ codon usage bias [12–15]. Bioinformatics methods analyses have shown that translation selection is probably the original reason for the formation of codon usage bias. Other possible factors affecting codon usage bias among species include: gene expression level [16], gene length [8], GC content [17], recombination rate, RNA stability [18], environmental stress [19], population size [20], evolutionary age of genes [21], and so on. Codon usage bias has profound influence on genomic evolution [22]. Even within the same genome, codon usage patterns are not necessarily the same within the same gene [23].

Envelope glycoprotein is a main fatty acid acylating glycoprotein of *Bombyx mori* nuclear polyhedrosis virus (BmNPV) [24]. With pH-dependent membrane fusion activity, it can make the virus and host cell fusion. Glycoprotein gene mainly is connected by disulfide bond into the form of trimer and exists in the end of stick baculovirus, forming a typical membrane-grain structure. Research shows that the monoclonal antibody of glycoprotein can make a significant reduction in the infectious virus particles [25]. The glycoprotein gene plays a key role in the progress that baculovirus infects cells and progeny nuclear capsid effective budding release [26, 27]. Besides, silencing glycoprotein gene in transgenic silkworm increases resistance to BmNPV [28, 29]. Therefore, the glycoprotein is one of the most important capsule membrane proteins in baculovirus. Because the glycoprotein gene product and its homologues are relatively conserved, it is an ideal gene useful in the study of the evolutionary relationships of different baculoviruses. Analyses of codon usage bias could therefore enable a better understanding of the NPV molecular evolution dynamic.

## Results

### Clustering analysis

**Fig. 1:**
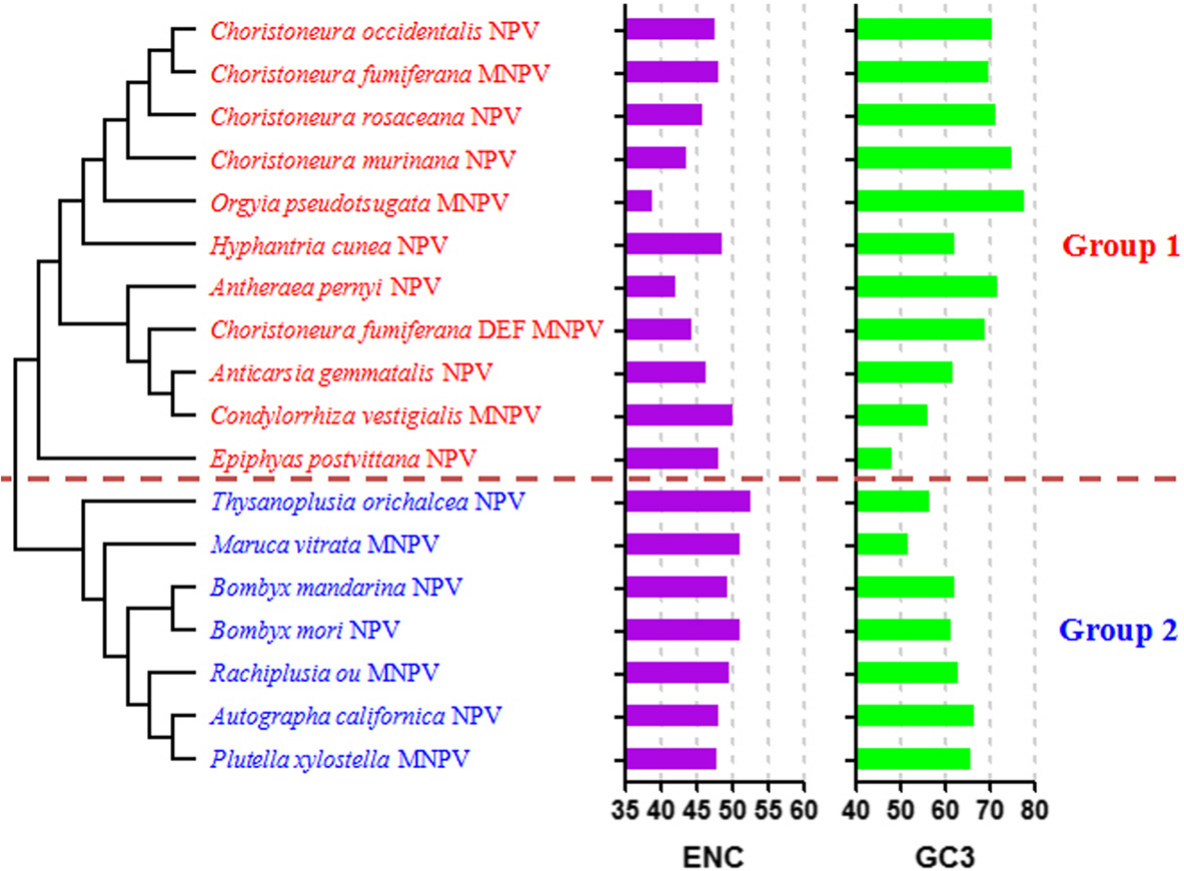
Neighbor-joining analysis of NPV species according to the glycoprotein gene. Effective number of codons and GC3 content for each species are also displayed

A total of 18 different NPV species were analyzed using a Neighbor-joining method specifically using their glycoprotein genes for calculation. The ENC and GC content in the third position (GC3) of triplet codons from each species are compared simultaneously. It is observed that *Choristoneura occidentalis* NPV(ChocNPV), *Choristoneura fumiferana* MNPV(CfMNPV), *Choristoneura rosaceana* NPV(CrNPV), *Choristoneura murinana* NPV(CmNPV), *Orgyia pseudotsugata* MNPV(OpMNPV), *Hyphantria cunea* NPV(HycuNPV), *Antheraea pernyi* NPV(AnpeNPV), *Choristoneura fumiferana* DEF MNPV(CfDEFMNPV), *Anticarsia gemmatalis* NPV(AgNPV), *Condylorrhiza vestigialis* MNPV(CvMNPV) and *Epiphyas postvittana* NPV(EppoNPV) belong to Group 1, indicating that their evolutionary relationship is similar. But, the variation range of their ENC value and GC3 content are more extensive (38.9 ≤ ENC ≤ 50.0 and 48.4 ≤ GC3 ≤ 77.5, respectively) (Fig. 1). This result shows that they do not exhibit similar codon usage bias although they possess equally evolutionary position. There are 7 NPV species with *Thysanoplusia orichalcea* NPV(ToNPV), *Maruca vitrata* MNPV(MvMNPV), *Bombyx mandarina* NPV(BmaNPV), *Bombyx mori* NPV(BmNPV), *Rachiplusia ou* MNPV(RaouMNPV), *Autographa californica* NPV(AcNPV) and *Plutella xylostella* MNPV(PlxyNPV) in Group 2. The variation range of their ENC value and GC3 content are relatively smaller (47.9 ≤ ENC ≤ 52.5 and 51.9 ≤ GC3 ≤ 66.7, respectively). It suggests that the closer the evolution of species classification, the more similar their codon usage bias.

**Fig. 2:**
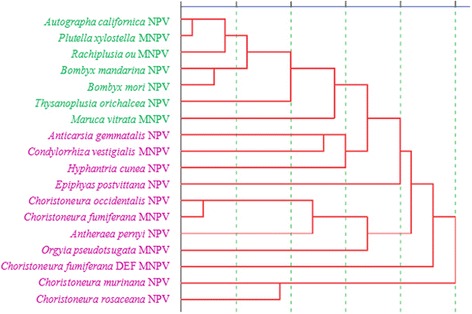
Cluster analysis of the 18 NPV species based on the RSCU values of glycoprotein. The 18 species were divided into two broad categories. Group 1 and Group 2 are shown in purple and green, respectively

On the other hand, 18 different NPV species were also analyzed using a cluster analysis method specifically using RSCU values for calculation. It is observed that AcNPV, PlxyNPV, RaouMNPV, BmNPV, BmaNPV, ToNPV and MvMNPV exhibit similar evolution status, consistent with the analysis results of Neighbor-joining (Fig. 2). Other species are similar to the results of Neighbor-joining on the whole, but still have some differences from the perspective of the individual evolutionary branch, such as AgNPV, CvMNPV and HycuNPV exhibit similar codon usage bias, indicating that the respective pairs are evolutionarily related.

It shows that there are some difference between Cluster and Neighbor-joining analysis, especially codon usage patterns are rather different in Group 2 but less apparent in Group 1.

### Glycoprotein codon usage bias of 18 NPVs analysis

We analyzed the glycoprotein genes of 18 NPV species. The GC content for these genes ranges from 42.7 to 54.4 %, with the average being 48.90 %. GC content varies most significantly in the first and third codon positions, with of values of 46.39 and 64.44 %, respectively. The ENC in glycoprotein varies from 38.9 to 52.5, with a mean of 47.39. Accordingly, none of the 18 glycoprotein genes exhibits strong codon bias, as all of their ENC values are above 35. This data shows that glycoprotein displays a general random codon usage, lacking strong codon bias (Table 1 and Additional file 1).

**Table 1:** Means and standard deviations of several index numbers from 18 glycoprotein genes in NPV

A3s (%)	T3s (%)	C3s (%)	G3s (%)	GC (%)	GC1 (%)	GC2 (%)	GC3 (%)	GC3s (%)	ENC	CAI
23.24 ± 5.01	25.24 ± 5.87	46.05 ± 5.45	37.70 ± 5.39	48.90 ± 3.13	46.39 ± 1.62	35.59 ± 1.05	64.44 ± 7.64	62.49 ± 7.98	47.39 ± 3.33	0.78 ± 0.01

Additionally, the relative synonymous codon usage (RSCU) values of 59 sense codons (except for Trp, initiator codon and terminator codon) also support this conclusion NPV glycoprotein presenting weak codon bias. Nearly half of the glycoprotein codons (24/59) are frequently used as shown in Table 2, such as GGC (coding Glycine) and UUG (coding Leucine). The RSCU values of our set of NPV glycoprotein genes were analyzed, as shown in Table 2. The result was representative of NPV as a whole. All of the UUU, UUG, CUG, AUU, GUG, AGC, UCG, CCC, CCG, ACC, ACG, GCC, GCG, UAC, CAC, CAA, AAC, AAA, GAC, GAG, UGC, CGU, CGC, and GGC codons have a somewhat high bias (RSCU > 1.0), esp UUU, UUG, AUU, GUG, AGC, CGC codons (RSCU > 1.5) possess a strong bias. Other codons are used less frequently, such as UUC, AUA, UCA, CCA, GCA, GAU, AGG, GGA, GGG, and etc. (RSCU < 1.0).

**Table 2:** The RSCU value and used codon numbers in the glycoprotein of NPV(9236 codons)

Amino acid	Codon	Number	RSCU	Amino acid	Codon	Number	RSCU
Phe	**UUU**	240	**1.57**	Ala	GCU	72	0.68
	UUC	65	0.43		**GCC**	168	**1.59**
Leu	UUA	74	0.59		GCA	28	0.27
	**UUG**	225	**1.80**		**GCG**	154	**1.46**
	CUU	84	0.67	Tyr	UAU	84	0.59
	CUC	112	0.90		**UAC**	202	**1.41**
	CUA	71	0.57	His	CAU	93	0.63
	**CUG**	183	**1.47**		**CAC**	203	**1.37**
Ile	**AUU**	336	**1.65**	Gln	**CAA**	141	**1.11**
	AUC	181	0.89		CAG	114	0.89
	AUA	95	0.47	Asn	AAU	182	0.51
Val	GUU	89	0.64		**AAC**	528	**1.49**
	GUC	95	0.68	Lys	**AAA**	367	**1.19**
	GUA	95	0.68		AAG	248	0.81
	**GUG**	278	**2.00**	Asp	GAU	149	0.46
Ser	AGU	73	0.69		**GAC**	497	**1.54**
	**AGC**	206	**1.94**	Glu	GAA	286	0.97
	UCU	64	0.60		**GAG**	306	**1.03**
	UCC	81	0.76	Cys	UGU	77	0.56
	UCA	42	0.39		**UGC**	200	**1.44**
	**UCG**	172	**1.62**	Arg	**CGU**	73	**1.01**
Pro	CCU	32	0.54		**CGC**	196	**2.72**
	**CCC**	74	**1.26**		CGA	39	0.54
	CCA	22	0.37		CGG	52	0.72
	**CCG**	107	**1.82**		AGA	41	0.57
Thr	ACU	103	0.68		AGG	32	0.44
	**ACC**	208	**1.37**	Gly	GGU	79	0.67
	ACA	94	0.62		**GGC**	321	**2.72**
	**ACG**	204	**1.34**		GGA	44	0.37
					GGG	28	0.24

Preferentially used codons are displayed in bold

**Fig. 3:**
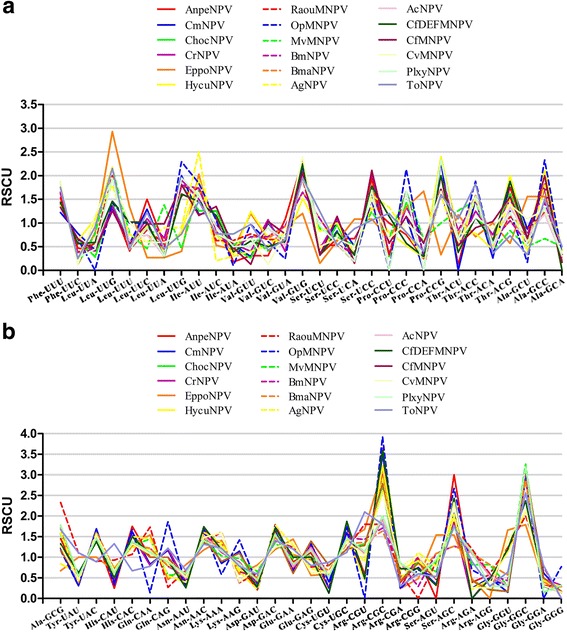
Analysis of relative synonymous codon usage of glycoprotein gene in 18 NPV species. **a** and **b** show the comparison of 59 sense codons from 18 NPV glycoproteins

Furthermore, we also compared the RSCU values of 59 sense codons (Fig. 3). There was some difference of the RSCU values of 59 sense codons from 18 NPVs glycoprotein, but the overall trend is relatively similar. This illustrates that relatively similar species maintain the stability codon usage patterns.

### Nucleotide composition affects the formation of codon usage bias

**Fig. 4:**
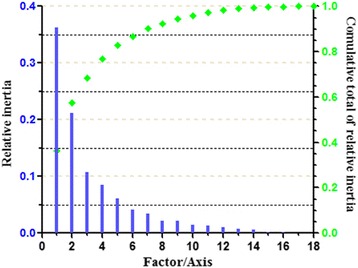
The correspondence analysis of the amino acid usage frequencies in glycoprotein. The relative and cumulative inertia of the first 18 factors were shown based on the correspondence analysis

Correspondence analysis was performed on the RSCU values. The axes factors, as shown in Fig. 4, are represented by Axis 1 and 2 which correlate to two main influencing factors of codon usage bias. They represent 36.29 and 21.20 % of the total variation, respectively (Fig. 4). The relationship between codon usage bias and amino acid composition were explained by multifactor variable analysis. Axis 1 has a distinct positive correlation with C3s (*r* = 0.965, *p* < 0.01), G3s (*r* = 0.948, *p* < 0.01), and GC3s (*r* = 0.996, *p* < 0.01). Axis 1 shows evidently negative correlation with A3s (*r*= −0.957, *p* < 0.01) and T3s (*r*= −0.969, *p* < 0.01). There is an obvious negative correlation between GC3s and ENC (*r* = −0.822, *p* < 0.01). However, GC3s exhibits a significantly positive correlation with Axis 1 (*r* = 0.996, *p* < 0.01). On the other hand, ENC shows significant negative correlation with Axis 1 (*r* = −0.806, *p* < 0.01) (Table 3). There is a high correlations among these parameters because their *R* value is greater than 0.8. These results demonstrate that nucleotide composition indeed affects codon usage bias.

**Table 3:** Correlation coefficients between the position of genes along the first two major axes with index of glycoprotein genes’ codon usage and synonymous codon usage bias

	Length	GC	GC1	GC2	GC3	GC3s	A3s	T3s	C3s	G3s	ENC	CAI	Axis1
GC	−0.353												
GC1	−0.498*	0.818**											
GC2	−0.672**	0.626**	0.730**										
GC3	−0.208	0.944**	0.685**	0.376									
GC3s	−0.208	0.944**	0.685**	0.376	1.000**								
A3s	0.207	−0.901**	−0.601**	−0.334	−0.959**	−0.959**							
T3s	0.283	−0.961**	−0.754**	−0.455	−0.973**	−0.973**	0.913**						
C3s	−0.308	0.973**	0.771**	0.502*	0.971**	0.971**	−0.913**	−0.979**					
G3s	−0.099	0.866**	0.571**	0.211	0.946**	0.946**	−0.973**	−0.911**	0.897**				
ENC	0.491*	−0.880**	−0.722**	−0.609**	−0.822**	−0.822**	0.763**	0.858**	−0.899**	−0.709**			
CAI	0.485*	−0.229	−0.142	−0.364	−0.154	−0.154	0.183	0.209	−0.186	−0.158	0.142		
Axis1	−0.198	0.938**	0.688**	0.378	0.996**	0.996**	−0.957**	−0.969**	0.965**	0.948**	−0.806**	0.361	
Axis2	0.760**	−0.152	−0.334	−0.671**	0.007	0.007	−0.032	0.044	−0.110	0.203	0.370	0.152	0.018

** *p* < 0.01. * *p* < 0.05

**Fig. 5:**
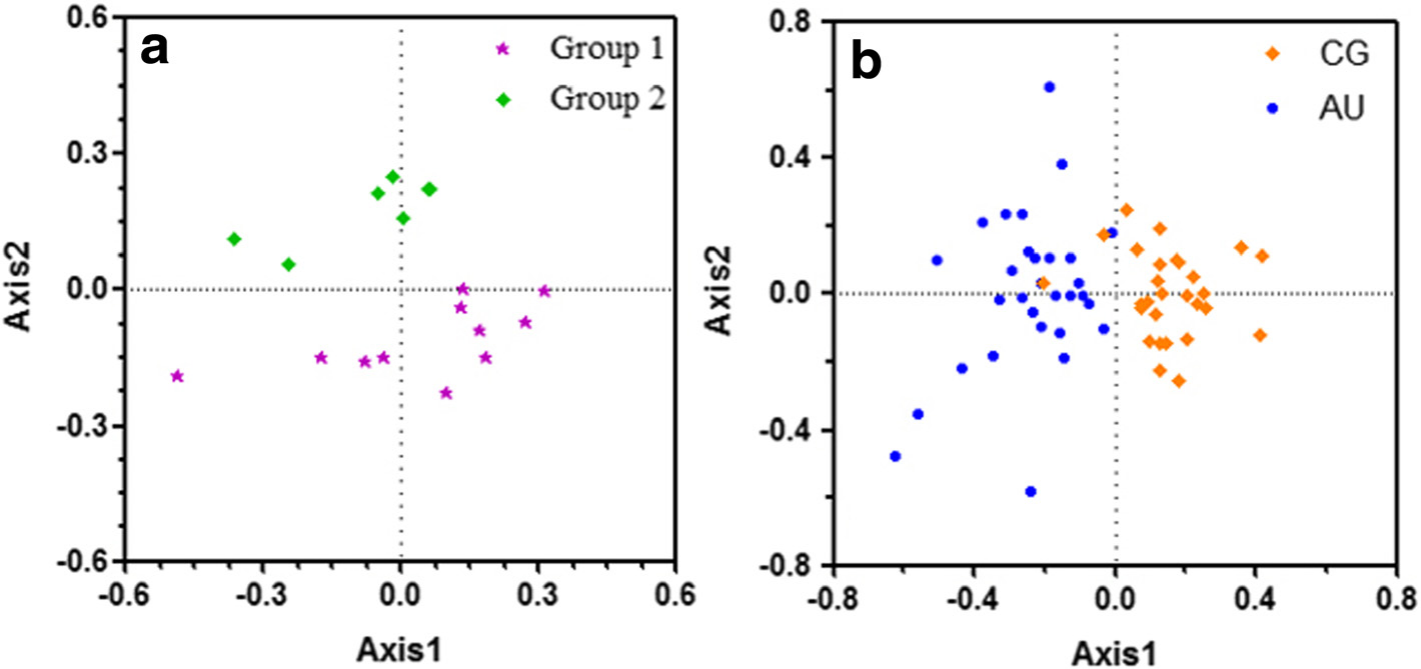
Correspondence analysis of RSCU for the total genes in NPV. **a** Distribution of the 18 glycoprotein genes in NPV on the plane corresponding to the coordinates on the first and second principal axes. *Purple star* and *green rhombus* indicate Group 1 and Group 2 NPV genes, respectively. **b** Correspondence analysis of the synonmous codon usage towards the codon in glycoprotein. Codon ending with C/G and A/U is shown in *orange rhombus* and *blue round*, respectively

All of the genes are diffusely distributed, and it indicates that many factors affect codon usage bias (Fig. 5a). Axis 1 represents the main index for affecting codon usage bias. The distribution density of triplet codons ending with G/C is closer to Axis 1 than that of codons ending with A/U (Fig. 5b). Thus, these results suggest that nucleotide composition (especially G and C) posits a certain degree of influence on the codon usage bias. Furthermore, the mutation impact of codons ending with G/C on codon usage bias is greater than that of codons ending with A/U.

### GC3s affecting codon bias

**Fig. 6:**
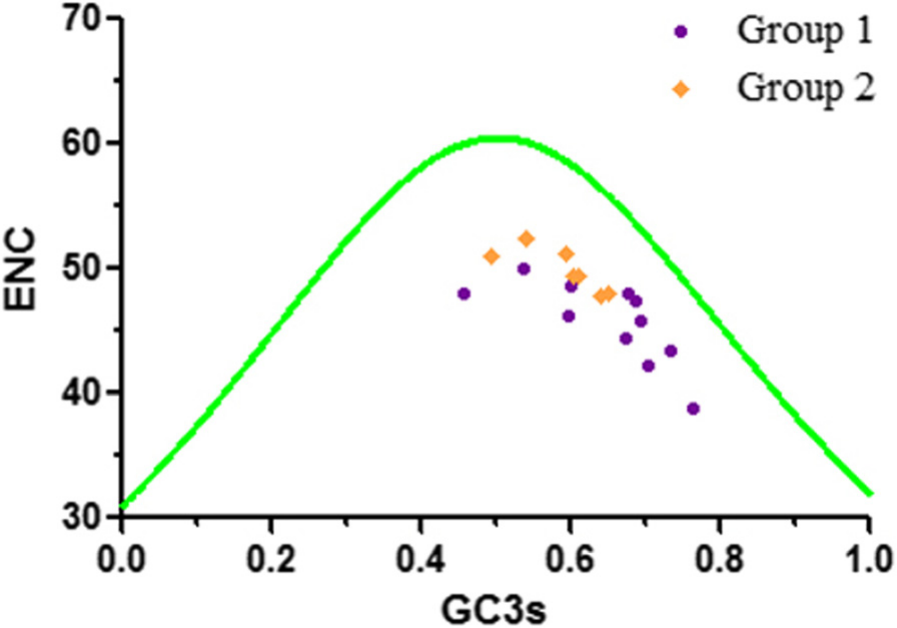
ENC values plotted against GC3s. The standard curve (*green line*) represents the relationship between ENC and GC3s under mutational pressure. ENC denotes the effective number of codons, and GC3s denotes GC content in the third synonymous codon position. Point on or near the curve means indicate bias caused by mutation pressure. Points beneath the curve indicate bias influenced by natural selection or other factors. NPV genes of Group 1 and Group 2 are shown in *purple round* and *orange rhombus*, respectively

A standard curve evaluates the relationship between ENC and GC3s, which illustrates their corresponding relationship of the extent under mutation pressures. If the points, which represent various genes, fall on or near the standard curve, the codon usage bias would be interpreted as being mainly determined by mutation pressures. Generally, codon usage bias depends on the content of the ending base in codons—in other words, the GC3s content of genes. However, all of the points (no matter the Group 1 or Group 2) are located beneath the standard curve, indicating that mutation pressure which is not the critical factor in the formation of codon preferences (Fig. 6). Thus, the GC3s values of glycoprotein are not the sole factors affecting codon bias formation in various species of NPV. Furthermore, the dispersed plotted genes indicates that other factors can impact codon usage bias to a certain extent. These factors include natural selection, gene length, and gene expression levels.

### Natural selection plays an important role in the process of codon bias formation

**Fig. 7:**
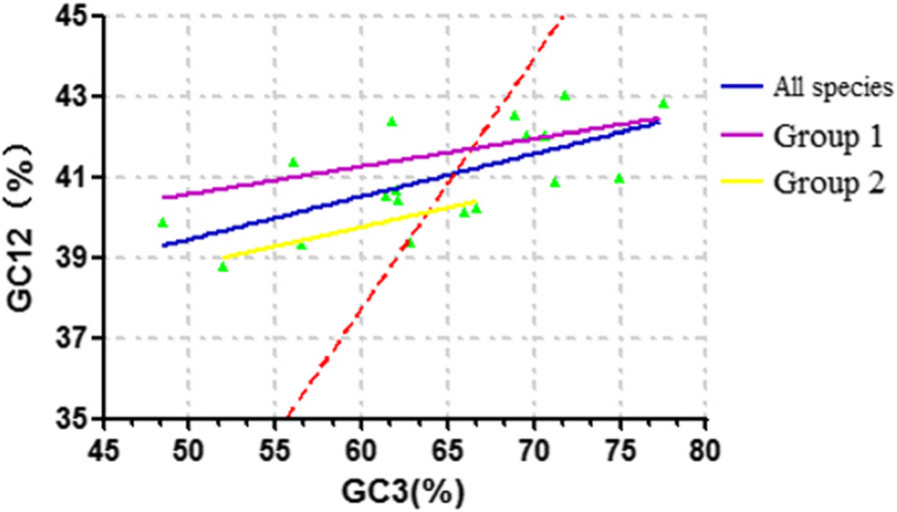
Neutrality plot analysis of the 18 glycoprotein genes. Neutrality plot analysis of the average GC content in the first and second positions of the codons (GC1 and GC2) and of the GC content in the third position (GC3) for glycoprotein. The *blue*, *purple* and *yellow* regression curve represented as y = 0.1063x + 34.14, R^2^ = 0.4307, y = 0.0685x + 37.15, R^2^ = 0.3517, y = 0.0956x + 34.02, R^2^ = 0.5645. The *diagonal line* is colored in *red dotted line*

ENC-plot analysis demonstrated the extent to which mutational pressures affect the formation of codon usage bias. We next seek to determine whether natural selection or mutation pressure plays a greater role in generating codon usage bias. To determine this, we attempt to carry out a neutrality plot analysis on the GC content of codons. The distribution range of GC3 is very broad, from 48.4 to 77.5 % (Fig. 7). There is indeed obvious correlation between GC1 and GC3 (*p* < 0.01), which initially seemed indicative of mutation pressure playing a greater role in direct codon usage bias. However, after calculating the neutrality plot, this was not the case. In Fig. 7, all of GC3 values diffuse distribution and all of regression curve deviate from the diagonal line. And then, the slope of the regression line was determined to be 0.1063, 0.0685 and 0.0956. Should the slope be equal to one (diagonal line), indicating a perfect correlation between GC12 and GC3, mutation pressure would be deemed the dominant factor in generating bias. Slopes approaching the vertical or horizontal axes would indicate natural selection as dominant. Despite the observed GC12 and GC3 correlation, our slope of 0.1063, 0.0685 and 0.0956 indicates that the influence of direct mutation pressure for codon usage bias is only 10.63, 6.85 and 9.56 %, respectively. The influence of natural selection on codon usage bias was calculated to be 89.37, 93.15 and 90.44 %, thereby indicating natural selection as the dominant factor influencing bias.

### Effects of gene length and expression level on codon usage bias

CAI values are useful in predicting the levels of gene expression. Silkworm ribosomal genes, which have a high level of expression, were used as references in our computation of codon adaptation indices [30]. Correlation analysis shows that CAI and ENC demonstrate no significant correlation, as well as no obvious correlation exists between the CAI, GC3s and GC content. This illustrates that gene expression levels have no effect on codon bias. On the other hand, gene length has no obvious correlation with CAI, ENC and Axis 1. This observation indicates that there is no correlation between the length of the gene and its codon usage bias for NPV glycoprotein.

**Fig. 8:**
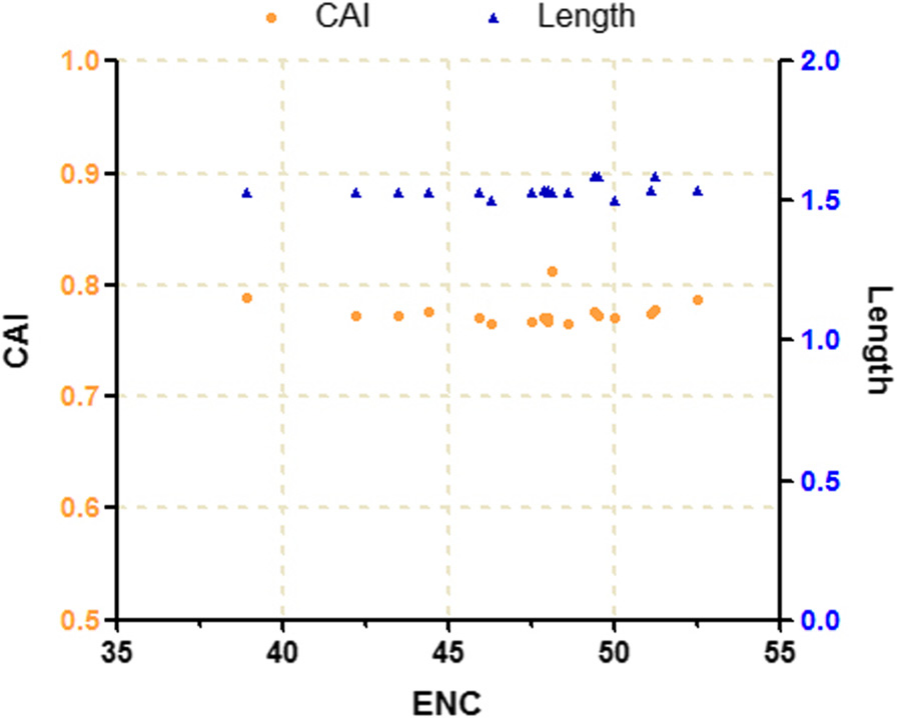
ENC values plotted against CAI and Length. The *orange round* represented the ENC value against CAI value for each gene, the *blue triangles* indicated the ENC value against Length value for each gene

The CAI values of the various glycoprotein genes ranges from 0.765 to 0.812, and the length of the gene ranges from 1500 to 1593 bp. The level of variation in CAI values and gene length among the various glycoprotein genes is relatively small, as shown in Fig. 8. These results indicate that gene expression level and length play an acute role in the shaping of codon bias. The gene lengths of the various viral species are all relatively similar, and given that all species CAI values are very approximate. It was suggested that the glycoprotein gene displays stable expression in the process of evolution in NPV.

## Discussion

Our clustering analysis statistics are similar to Neighbor-joining. We compared the RSCU values of glycoprotein from 18 species, the results show that they have relative similarity codon usage bias. After a series of analyses, glycoprotein possess a general codon usage pattern because all the ENC values are greater than 35. RSCU values are an index for assessing frequency of synonymous codon usage. RSCU = 1.0 means that there is only one codon within a synonymous codon set, and it indicates that the codon is not biased. Alternatively, RSCU > 1.0 indicates a high frequency bias for a particular codon within a synonymous codon set, and vice versa [31]. Many factors can result in the synonymous codon usage bias of the glycoprotein gene of the NPV genus. Nucleotide composition is one of the factors that affect codon usage bias especially codons ending with G/C. But, most of the codons ending with A/U also demonstrated a stronger frequency of codon usage bias in other species that contains rich A/T base pairs, such as *Saccharomyces cerevisiae* and *Plasmodium falciparum* [32, 33]. In addition, previous studies have identified mutational pressures and natural selection as two major factors influencing codon usage bias [34]. The ENC-plot is an effective tool for measuring codon usage bias [35]. Our ENC-plot analysis showed that mutational pressures can slightly affect the formation of codon usage bias. However, our neutrality plot analysis indicates that natural selection might play an important role in shaping the codon usage bias. This phenomenon also exists in other species, such as *Arabidopsis thaliana*, *Drosophila melanogaster,* and *Caenorhabditis elegans*, in which natural selection is also highly important in shaping codon usage bias in the complete genome [36]. Through previous research we know that *gp64* is a highly conserved gene [37]. It is also one of the homologue of NPV glycoprotein. According to the results of this study, the gene length of 18 glycoprotein is almost the identical size, and their CAI values are maintained at the same level. These results illustrate that the length of the glycoprotein and its expression level would not random variation, in other words, glycoprotein is one of the conservative genes in NPV.

## Conclusions

Codon usage patterns were similar between different NPV viral species in same genus. Both Neighbor-joining analysis and clustering analysis were showing the similar conclusion. Multiple factors can affect the synonymous codon usage bias of every organism. Through a series of research and analysis, we can draw the following conclusions: The glycoprotein gene of the NPV genus exhibits a weak codon usage bias. Nucleotide composition, mutation pressure, gene length, and gene expression levels all influence synonymous codon usage bias, with natural selection being the main influence factor. Though codon usage bias is not a necessary metric for carrying out traditional phylogenetic analysis, our study enables us to understand the molecular and genetic mechanisms of viral evolution from a novel perspective. Future advances in the understanding of codon usage evolution will undoubtedly aid us in achieving a more nuanced mastery of viral genetics.

## Methods

### Codon usage bias measurement index

The effective number of codons (ENC) is a measure that quantifies the extent to which the usage of a gene departs from the equal usage of synonymous codons. It is an excellent indicator of codon usage bias in both genes and genomes. The minimum ENc value is 20, indicating severe codon usage bias, and the maximum value is 61, indicating equally likely usage of all codons.

Relative synonymous codon usage (RSCU) refers to a relative ratio that describes the usage frequency of one specific codon compared to the usage frequency of synonymous codon for the same corresponding amino acid. If the RSCU value is 1, codons are used equally with no bias. Codons with an RSCU value greater than 1 exhibit strong bias (i.e., used more frequently than other synonymous codons), whereas codons with an RSCU value less than 1 exhibit negative bias and are used less frequently than other synonymous codons.

The codon adaptation index (CAI) is another effective measure of codon usage bias, in which each codon is referenced to an optimal codon frequency derived from a set of highly expressed genes. CAI values range from 0 to 1. A value of one indicates strong codon bias in which the optimal codon is always used, and vice versa.

### Multifactor variable analysis

Correspondence analysis (COA) is a widely used statistical method used in the analysis of multiple factors and their influences on a particular component. With respect to our experiment, correspondence analysis was used to analyze the effects of various factors on the formation of synonymous codon usage bias in various genes.

Linear regression analysis (LRA) and factor analysis (FA) were used to analyze the relationship between the ENC values and GC3 content of glycoprotein in NPV and the level of correlation between GC12 content and GC3 content. This analysis allowed us to deduce the effects of mutational pressure on codon bias formation.

Neighbor joining (NJ) is a bottom-up clustering method for the creation of phylogenetic trees. Usually used for trees on DNA or protein sequence data, the algorithm knowledge of the distance between each pair of taxa to form the tree.

Cluster analysis (CA) is an analytical method that divides data into groups in such a way that elements more similar to each other are grouped together. Distance is not constant in cluster analysis. The euclidean distance which describes the linear correlation between two variables, was used in our analysis to determine distance.

ENC-plot analysis was used to determine the decisive factors affecting codon usage bias. Each point in the plot corresponds to a GC3s value of a particular gene. Sets of points located on the standard curve indicate mutational pressure determines codon usage bias. Alternatively, points located below the standard curve indicate there are other factors other than mutational pressure affecting codon usage bias.

A neutrality plot analysis was used to determine the extent to which mutational pressures affect codon usage bias as compared to natural selection. Synonymous codon mutations often occur in the third position of the codon, though at times mutations may also occur in the first and second positions, leading to non-synonymous codons. Using GC3 as a horizontal coordinate and GC12 as a vertical coordinate, the GC3 and GC12 contents of glycoprotein genes were plotted and a regression line was calculated to determine the extent to which mutational pressures played a role in the formation of codon usage bias as opposed to natural selection. Regression lines that fall near the diagonal (slope = 1) indicate weak external selection pressures on the generation of codon usage bias, whereas regression curves deviating from the diagonal indicate a heavy influence of natural selection on codon usage bias.

### Software

All indices of codon usage bias above were calculated from the data set using the program CodonW 1.4.4 (http:// codonw.sourceforge.net/). Clustering analysis and correlations between codon usage variations amongst indices of codon usage were carried out using a statistical software called SPSS Version 22.0, MEGA 6.0, ClustalX 2.0 and GraphPad Prism 5.0.

## Additional file

Additional file 1: Table S1. All the indices of total genes. (XLS 27 kb)

## Abbreviations

A3s: A content on the third synonymous codon position
C3s: C content on the third synonymous codon position
CAI: Codon adaptation index
COA: Correspondence analysis
ENC: Effective number of codons
FA: Factor analysis
G3s: G content on the third synonymous codon position
GC1: GC content in the first position of the codons
GC12: Stands for the average value of GC content in the first and second position of the codons
GC2: GC content in the second position of the codons
GC3: GC content in the third position
GC3s: GC content on the third synonymous codon position
LRA: Linear regression analysis
RSCU: Relative synonymous codon usage
T3s: T content on the third synonymous codon position
